# The external evocation and movement-related modulation of motor cortex inhibition in children and adolescents with Tourette syndrome – a TMS/EEG study

**DOI:** 10.3389/fnins.2023.1209801

**Published:** 2023-10-19

**Authors:** Julia Schmidgen, Kerstin Konrad, Veit Roessner, Stephan Bender

**Affiliations:** ^1^Department of Child and Adolescent Psychiatry, Medical Faculty and University Hospital, University of Cologne, Cologne, Germany; ^2^Child Neuropsychology Section, Department of Child and Adolescent Psychiatry, Psychosomatics and Psychotherapy, University Hospital, RWTH Aachen, Aachen, Germany; ^3^JARA-BRAIN Institute II, Molecular Neuroscience and Neuroimaging, Forschungszentrum Jülich GmbH and RWTH Aachen University, Jülich, Germany; ^4^Department of Child and Adolescent Psychiatry, Faculty of Medicine Carl Custav Carus, TU, Dresden, Germany

**Keywords:** Tourette syndrome, inhibition, TMS, EEG, N100, MEP

## Abstract

**Objective:**

This study tested the reactivity of motor cortex inhibition to different intensities of external stimulation by transcranial magnetic stimulation (TMS) and its internal modulation during different motor states in children and adolescents with Tourette syndrome.

**Methods:**

TMS-evoked N100 served as an indirect measure of GABA_B_ receptor function which is related to cortical inhibition. Combined TMS/EEG was used to analyze the TMS-evoked N100 component evoked by different stimulation intensities as well as during resting condition, movement preparation (contingent negative variation task) and movement execution. The study included 18 early adolescents with Tourette syndrome and 15 typically developing control subjects.

**Results:**

TMS-evoked N100 showed a less steep increase with increasing TMS intensity in Tourette syndrome together with less modulation (disinhibition) over the primary motor cortex during the motor states movement preparation and movement execution. Children with Tourette syndrome showed equally high N100 amplitudes at 110% resting motor threshold (RMT) intensity during resting condition and a parallel decline of RMT and N100 amplitude with increasing age as control subjects.

**Conclusion:**

Our study yields preliminary evidence that modulation of motor cortical inhibitory circuits, during external direct stimulation by different TMS intensities and during volitional movement preparation and execution is different in children and adolescents with Tourette syndrome compared to controls. These results suggest that a reduced resting motor cortical inhibitory “reserve” could contribute to the production of unwanted movements. Our findings are compatible with increased regulation of motor cortex excitability by perception-action binding in Tourette syndrome instead of top-down / motor regulation and need to be replicated in further studies.

## 1. Introduction

Tourette syndrome (TS) is a complex neurodevelopmental childhood-onset condition characterized by the co-occurrence of multiple motor and at least one vocal tic over the period of minimum 1 year. Although the underlying mechanism of TS is currently poorly understood, evidence suggests functional impairments within the basal ganglia and several parallel cortico-striato-thalamocortical circuits. However, it remains unclear, which components within the pathways contribute to tics, which may be regarded as a surplus of actions. Some studies indicate that multiple sources within the circuits lead to a divergent input to the primary motor cortex. Motor cortical areas might be hyperexcited due to a reduced inhibitory input to the motor cortex, as shown by transcranial magnetic stimulation (TMS) studies. However, various studies showed contradictory findings regarding tic-related pathophysiological mechanisms.

Especially animal model data and magnetic resonance spectroscopy studies have indicated a major role of differences in the glutamate (excitatory) and γ-aminobutyric acid (GABA) neurotransmitter system (inhibitory) in tic pathophysiology. GABA-ergic neurotransmission plays a crucial role in the regulation of neuronal activity during various states of motor activation. During the resting state it ensures a constant level of neuronal activity and prevents uncontrolled generation and spreading of excitatory signals. During movement preparation and execution, regulation of GABAergic inhibition (Nowak et al., 2017) modulates the excitability of motor circuits to ensure an efficient and controllable movement execution (Dupont-Hadwen et al., 2019). Preparatory excitation of motor networks prior to a movement enables the fast transmission of neuronal signals, effective muscle activation and enhanced precision due to suppression of competing motor areas. However, excessive excitability during motor facilitation, execution, or resting state, could cause uncontrolled, premature, or inefficient movements. Regarding TS, post-mortem investigations showed a reduced number and altered distribution of inhibitory GABAergic interneurons within the sensorimotor areas of the striatum (Kalanithi et al., 2005; Kataoka et al., 2010). Multiple paired-pulse TMS studies consistently reported diminished GABA_A_-mediated intracortical inhibition within the TS motor cortex (Gilbert et al., 2004; Orth et al., 2008; Orth and Rothwell, 2009; Heise et al., 2010). Reduced GABA-mediated motor cortical inhibition has frequently been interpreted as a core pathophysiological mechanism contributing to the generation of tics (Jackson et al., 2015).

TS usually reaches its maximum severity in early adolescence. Afterwards most TS patients experience a considerable improvement of the symptoms, characterized by a diminution of intensity and frequency of tics in late adolescence or early adulthood. Compensatory mechanisms are thought to contribute to an increased control over the motor output and concomitant over tics due to an elevated tonic inhibition. TMS-based findings have been interpreted as reduced gain within motor cortical circuits, which could represent a secondary consequence of or adaptation to TS. In this sense, deficits in inhibitory circuits in children and adolescents with TS might be compensated by reducing the gain in corticospinal excitability. Consequently, this would lead to decreased sensitivity to changes in input from other brain areas or external stimuli (Schilke et al., 2022).

Besides deficits in GABA_A_-mediated inhibitory circuits, GABA_B_-mediated circuits might also be deficient within the TS motor cortex. According to this notion, evidence from human TMS (Sanger et al., 2001), human pharmaco-TMS (McDonnell et al., 2006), and animal studies (Pitler and Alger, 1994; Deisz, 1999) suggest that activation of presynaptic GABA_B_ receptors may halt release of GABA. Even though GABA_A_-mediated motor cortical inhibition is evidently deficient in TS, the influence of GABA_B_-mediated intracortical inhibition has been studied rarely. Previous studies have reported inconsistent results and focused mainly on long interval intracortical inhibition (LICI) as a measure of GABA_B_-mediated inhibition at rest (Ziemann et al., 1997; Gilbert et al., 2004; Orth et al., 2008).

Combined TMS/EEG studies have highlighted the possibility of directly assessing primarily GABA_B_-mediated motor cortical inhibition (Ilmoniemi and Kičić, 2010; Daskalakis et al., 2012). This has been verified by Premoli et al. (2014) in a pharmaco-TMS-EEG study. Their study showed that baclofen, a GABA_B_ receptor agonist, specifically increased the TMS-evoked N100 amplitude, whereas Alprozam and Zolpidem, GABA_A_ receptor positive agonists, exerted a diminishing of the component or no effect. There is a body of evidence showing a strong relation between the TMS-evoked N100 and LICI as a measure of GABA_B_-mediated cortical inhibition in humans (Rogasch et al., 2013). Therefore, as in recent TMS/EEG research (Farzan et al., 2013; Rogasch et al., 2013; Premoli et al., 2014), it has been suggested that the TMS-evoked N100 is the most effective TMS measure of GABA_B_-mediated motor cortical inhibition (Rogasch et al., 2013). Since TMS evoked N100 component amplitudes were shown to be reduced during movement execution (Nikulin et al., 2003) and preparation (Bender et al., 2005; D’Agati et al., 2014), several findings further imply that the TMS-evoked N100 is also a functional marker of motor cortical inhibition.

To our knowledge, TMS/EEG has never been used to assess cortical inhibition through the analysis of TMS-evoked N100 component in TS. More importantly, the dependence of motor cortical deficits on specific motor cortical activity states (motor states) in early adolescent TS has not yet been investigated. The present study examined how motor cortex inhibition depends on top down-modulation by other cortical and subcortical areas during distinct motor states. In addition, we examined the responsiveness and modulatory capacities of cortical inhibition to different intensities of external direct stimulation by TMS. This way, two types of modulation of motor cortex inhibition could be examined, which are both independent from modulation by sensory input similar to an “urge,” though movement execution includes reafferent sensory feedback. In 18 early adolescent TS patients and 15 control subjects, we investigated motor cortical inhibitory processes associated with GABA_B_-mediated inhibition by the analysis of the TMS-evoked N100 component using combined TMS/EEG. Participants performed three different tasks, each aimed to examine a specific motor state, i.e., rest, movement preparation (forewarned reaction time task), and movement execution. Less reactivity to external stimulation together with less movement-related modulation of motor cortex inhibition could point towards lower inhibitory capacities and differences in the modulation of motor cortical excitability in TS, so that unwanted activity in “tic generator” circuits would pass the threshold to involuntary movements more easily (Heise et al., 2010).

## 2. Methods

### 2.1. Subjects

Eighteen adolescent subjects with a current diagnosis of TS were included from the outpatient TS clinic of the Department of Child and Adolescent Psychiatry, Dresden. Patients fulfilled DSM 5 criteria for TS. The control group included 15 typically developing adolescents (control subjects). All subjects were right-handed (Oldfields Edinburgh Handedness Inventory, 1971) and of normal intelligence. Intelligence levels were assessed using a validated short version of the fourth edition of the Wechsler intelligence test (Waldmann, 2008). Groups did not differ with respect to age [*t*(31) = 0.79; *p* = 0.43], handedness [*t*(31) = 0.03; *p* = 0.98], IQ [*t*(31) = −1.28; *p* = 0.21], or gender distribution [*t*(31) = 1.09; *p* = 0.28; cf. Table 1].

**Table 1:** Demographic, clinical, and TMS sample measures.

	Controls	TS
Sample size	*N* = 15	*N* = 18
Age (years; mean ± SD; range)	12.2 (2.3; 8.2–15.8)	12.8 (2.0; 10.7–17.6)
Gender (male, female)	9 m, 6 f	14 m, 4 f
EHI (mean ± SD)	77.6 (19.1)	77.8 (15.1)
IQ (mean ± SD)	116.5 (8.9)	111.6 (12.6)
YGTSS total symptom score (mean ± SD)	-	23.3 (18.2)
Comorbid ADHD	-	6 (18)
Resting motor threshold (mean ± SD)	72.9 (9.3) % MSO	74.5 (12.6) % MSO

TS = Tourette syndrome; EHI = Edinburgh Handedness Inventory; YGTSS = Yale global tic severity score; ADHD = Attention deficit hyperactivity disorder, MSO = maximum stimulator output.

Both groups were screened for psychiatric disorders using the German version of the M.I.N.I KID (Sheehan et al., 2010). Comorbid disorders, except for attention deficit hyperactivity disorder (ADHD), were excluded from the study. Comorbid ADHD, present in six subjects with TS, was assessed using a validated German ADHD questionnaire (Brühl et al., 2000), that has shown reliability as well as factorial and convergent/discriminant validity comparable to the Conners’ scale (Erhart et al., 2008).

Current tic severity was assessed using the Yale global tic severity score interview (YGTSS) on the day of the testing (Leckman et al., 1989; Storch et al., 2005). Most subjects with TS were treatment naive, yet two subjects received tiapride and one subject received aripiprazole. All participants as well as their first-degree family members had no history of epilepsy or any kind of seizures. For sample characteristics, see Table 1. Informed written consent was obtained by all participants and their legal guardians in accordance with the declaration of Helsinki. The study was approved by the local ethics committee.

### 2.2. Transcranial magnetic stimulation

Biphasic single pulse TMS (PowerMAG research 100; MAG & More GmbH, DE) was applied using a standard figure-of-eight coil (196 mm × 100 mm × 13.5 mm). Resting motor threshold (RMT) of the left primary motor cortex was assessed and determined using a conventional protocol (Conforto et al., 2004; Wu et al., 2012). First, participants were familiarized with the TMS sensation at low stimulation intensity of 40%. The intensity was then increased in steps of 10% of the device’s maximum stimulator output (MSO) until the first motor evoked potential (MEP) was identified. MEPs were recorded from the right first dorsal interosseous (FDI) muscle.

Next, optimal target location and coil orientation was adjusted to elicit a well-formed, peak-to peak measured and reliable MEP. The left primary motor cortex was determined functionally at the site where the largest MEP was elicited. To ensure accuracy of targeting and orientation we employed navigated TMS. Neuronavigation served to control for coil displacement throughout the measurement including coil angle (to maintain stimulation constant on the functionally determined hot spot). Thus, individual head landmarks were matched with a dummy head model using Brainvoyager QX software (Version 2.3, Brain Innovation BV, NL). The optimal individual stimulation target point was then pinpointed on the standard head model. Hence, optimal coil orientation and placement was live monitored and adjusted when necessary.

Finally, the RMT was assessed by sequential 2% increments in intensity starting at intensity 20% below hot-spot determination until five out of 10 MEPs (peak-to-peak) of at least 50 μV were registered. Suprathreshold single pulse TMS was applied at 110% of participants RMT. In addition to RMT-adjusted stimulation, due to only limited correlations of TMS-evoked potentials and MEP amplitudes, an RMT-independent stimulus-intensity slope was measured by stimulation at 40, 60, and 80% MSO for all subjects. Note that this differs from the RMT-standardization in most studies. It has the advantage to avoid a masking of TEP recruitment by RMT (EMG-related) effects, as TEPs and MEPs are qualitatively different measures.

### 2.3. Electroencephalographic recordings

EEG activity was continuously recorded at 5 kHz sampling rate using 64 channel TMS-compatible EEG equipment (Brainamp DC, BrainProducts). The high sampling served to minimize the TMS-artifact duration. Online filtering was set at DC and 1 kHz high-cutoff. Equidistant electrode caps (Easycap GmbH) were fixed carrying 64 sintered silver/silver chloride electrodes. The size of the electrode caps was adjusted to head circumference. Recording reference was electrode “Fpz.” Electrode impedance level was kept below 5 kΩ. Vertical electro-oculogram was recorded from electrodes FP1 and one electrode attached 2 cm below the left eye. Two electrodes each 1 cm lateral to the outer canthi recorded the horizontal electro-oculogram. NBS Presentation software (Version 15.0, Neurobehavioral Systems Inc.) was used to send triggers to both the EEG recording system and the TMS device. Recorded EEG data were first processed offline using Brain Vision Analyzer (BrainProducts). As this study focused on late TEPs >50 ms latency, TMS artifacts were eliminated by means of linear interpolation of the interval 5 to 40 ms with respect to the TMS trigger (Fuggetta et al., 2006; Taylor et al., 2010). Due to long-lasting sine wave artifacts (the anti-aliasing filter turns amplifier saturation into sine wave artifacts) because of high stimulation intensities in children with higher resting motor thresholds, the interpolated period was longer than usual (Taylor et al., 2008). We assured, that later time intervals were not affected by TMS-artifacts (Bonato et al., 2006), comparing ICA-based correction to interpolated data in single subject averages. Next, data were down-sampled to 500 Hz and filtered (48 dB/Oct) using a 50 Hz notch filter, a time constant of 1 s and a high-cutoff filter of 25 Hz. Then, data were average referenced. Segments comprised 1 s duration, −500 to 500 ms with respect to the TMS pulse. A 100 ms interval, −130 to −30 ms, was used for baseline correction. Visual inspection revealed no time-locked activity between −30 and −5 ms, however, we wanted to exclude any possible effects of the filtering near the interpolation interval on the baseline. Next, ocular artifact correction (Gratton et al., 1983) was applied as implemented in Brain Vision Analyzer (BrainProducts) followed by an automatic artifact removal of the segmented data (max allowed voltage step = 50 μV/ms; max allowed differences of values in intervals = 300 μV). The results of this automatic procedure were controlled for by visual inspection by a research assistant blind to the study hypotheses. Corrected and remaining segments were averaged by subject and condition. The TEP at a latency of approximately 100 ms, i.e., the TMS evoked N100, was registered at electrode C3 since previous studies have shown N100 to peak over the stimulated primary motor cortex (Nikulin et al., 2003; Bender et al., 2005; Bruckmann et al., 2012). The TMS-evoked N100 was quantified as the area under the curve in the time interval 70–150 ms (mean amplitude * 80 ms), in order to equalize any latency differences and to consider not only the peak amplitude but also the duration of the N100 component.

### 2.4. Electromyographic recordings

Electromyographic activity was recorded (Brainamp ExG, BrainProducts) by electrodes placed in a belly-tendon montage at the contralateral first dorsal interosseous muscle (FDI) using silver/silver-chloride self-adhesive surface electrodes (Neuroline 700, Ambu). EMG was sampled at 5 kHz with a time constant of 10 s and a high cutoff of 1 kHz. Offline, data were downsampled to 500 Hz and high pass filtered at 20 Hz (48 dB/Oct). Segmentation and baseline correction were carried out identical to the EEG processing (see section EEG above). Next, data were averaged across subjects and conditions. MEPs were quantified as the peak-to-peak amplitude within 18–40 ms after the TMS pulse.

### 2.5. Experimental procedure

Participants were seated in a sound attenuated, dimly lit room, facing a 22-inch computer screen (Fujitsu B22W-7 LED, 1680 × 1050 resolution). The sequence of the three experimental tasks was counterbalanced to obtain 18 individual sequences. Each sequence was randomly assigned to one participant of each group. The counterbalancing was imperfect due to the size of our sample. This however did not exert a significant confounding influence when we tested for order effects statistically. Experimental paradigms were implemented using NBS Presentation software (Version 15.0, Neurobehavioral Systems Inc.). Subjects were seated at 0.7 m distance to the PC screen. The default computer screen showed a vertically and horizontally centered white fixation cross (font size = 36) on a dark grey background and served to minimize eye movement. To reduce head movements and the risk of neck strain due to the TMS coil weight, subjects placed their heads on a cushioned, custom-made chin rest. To minimize TMS related acoustic evoked potentials participants wore earplugs. Every task started with an instruction presented in white font on the default background, followed by five rehearsal trials to ensure task comprehension. No acoustic masking by white noise was employed (Jarczok et al., 2021; Roos et al., 2021) as this masking procedure is not well tolerated in children and TMS-evoked N100 differs largely in frequency (duration), lateralization and amplitude from an auditory N1 in the examined age range. In contrast to the analyzed TMS-evoked N100 component, developmental AEP data showed that frontocentral N1b increases in children and adolescents and that the evoked peak is less broad and shows lower amplitudes (Bender et al., 2006). We applied 20 TMS pulses for each motor state condition. Due to larger TEP amplitudes in children (Bender et al., 2005) and adolescents, fewer trials are sufficient than in adults (D’Agati et al., 2014; Jarczok et al., 2016), especially because children do not tolerate long recording times with larger numbers of trials.

### 2.6. Experimental conditions – 3 motor states: rest, preparation, movement execution, and reactivity to external stimulation at different intensities at rest

#### 2.6.1. At rest (motor state 1)

Participants were instructed to rest, look at the fixation cross and neglect the occasional TMS sensation. Twenty single TMS pulses at 110% RMT were applied at an inter-trial-interval that randomly varied between 6 and 10 s. The inter-trial-interval was within the same range for all tasks (motor states 1 to 3).

#### 2.6.2. Motor preparation (motor state 2)

The task consisted of 20 trials of a contingent negative variation (CNV) paradigm, starting with a visual warning stimulus S1 (white exclamation mark, size = 34 × 27 mm) presented for a duration of 150 ms. An imperative stimulus S2 (white outline of a right hand, size = 34 × 27 mm) was presented for 150 ms, 3.3 s following S1 onset. Participants were instructed to prepare, at occurrence of S1, and to respond as quick as possible by clicking the left mouse button with the index finger of their right hand upon presentation of S2. Trials were terminated by the button press and followed by the inter-trial-interval. The 20 TMS pulses at 110% RMT occurred 2.8 s after S1 onset, to probe advanced motor preparation.

#### 2.6.3. Motor execution (motor state 3)

Participants were asked to trigger 20 TMS pulses with an intensity of 110% RMT in a self-paced manner by clicking the left mouse button with the index finger of their right hands. During task execution, i.e., 20 mouse clicks, participants were instructed to look at the fixation cross of the default screen and to produce self-paced clicks without any rhythm, rapid sequences or response pattern.

#### 2.6.4. Reactivity to external stimulation at different intensities

At rest, 20 TMS pulses were applied for 40, 60, and 80% maximum stimulator output (equal conditions as motor state 1).

### 2.7. Statistical analysis

All statistical analysis were performed using Statistica. Statistical significance level was determined as alpha = 0.05. Age-dependent development of RMT and N100 amplitude were compared between the two diagnostic groups in linear models. General linear models with the intersubject factor diagnostic group (TS versus control subjects), gender (male, female) as well as the repeated measurement factors modulation TYPE (internal modulation by motor state vs. external stimulation/intensity slope), each at two different INTENSITIES (internal: difference rest – motor preparation, difference rest – motor execution; external stimulation: difference 40–60% and 60–80% MSO) were calculated for N100 amplitude modulation between the conditions (N100 amplitude differences) with the linear predictors age and N100 amplitude at rest (110% MSO). The classification into “small” and “large” intensities refers to the amount of modulation, i.e., the size of the difference of TMS-evoked N100 amplitude in this condition compared to the resting condition. Raffin et al. (2020) showed that an increase in small stimulation intensities (40 to 60% RMT) had a lower effect on TEP amplitudes than the same increase in larger stimulation intensities, closer to the resting motor threshold (60 to 80% RMT). In the same line, it was shown that TMS-evoked neuronal activity increases in a sigmoid-shaped stimulus-intensity curve with a stronger modulatory effect for higher stimulation intensities around the RMT (Komssi et al., 2004). With regard to movement states, previous studies showed that the effect size of movement preparation on the TMS-evoked N100 (Bender et al., 2005) is lower than the effect size of movement execution (Nikulin et al., 2003). Though in these studies a RMT-standardization of the TMS-intensities has been performed, we believe that this fact does not qualitatively change the sigmoid recruitment curves. In order to analyze motor cortical inhibition modulation in general and to avoid multiple testing, the modulation types were included in one model. A main or interaction effect involving modulation TYPE would point towards specific effects of external and top-down modulation. Levene’s test did not detect any violation of the assumption of variance homogeneity. Note that due to the non-linear slope of the input–output curve (steeper slope around the RMT 60 vs. 80% MSO than for the subthreshold intensities 40 vs. 60% MSO), the difference 60 vs. 80% MSO was larger than 40 vs. 60% MSO, though the TMS-evoked N100 rises already at lower intensities than the MEP (cortical response before EMG response). MEP and N100 amplitudes at 110% RMT were compared between the two diagnostic groups, correcting for age and gender.

## 3. Results

### 3.1. Behavioral performance during motor state 2 (motor preparation)

Adolescents with TS showed comparable task performance to control subjects regarding reaction times in the motor preparation (motor state 2) task paradigm (Controls: 161 ± 36 ms; TS: 150 ± 33 ms). Due to the small number of TS subjects, comorbid ADHD did not show any covariate effects on performance.

### 3.2. Cortical inhibition

**Figure 1:**
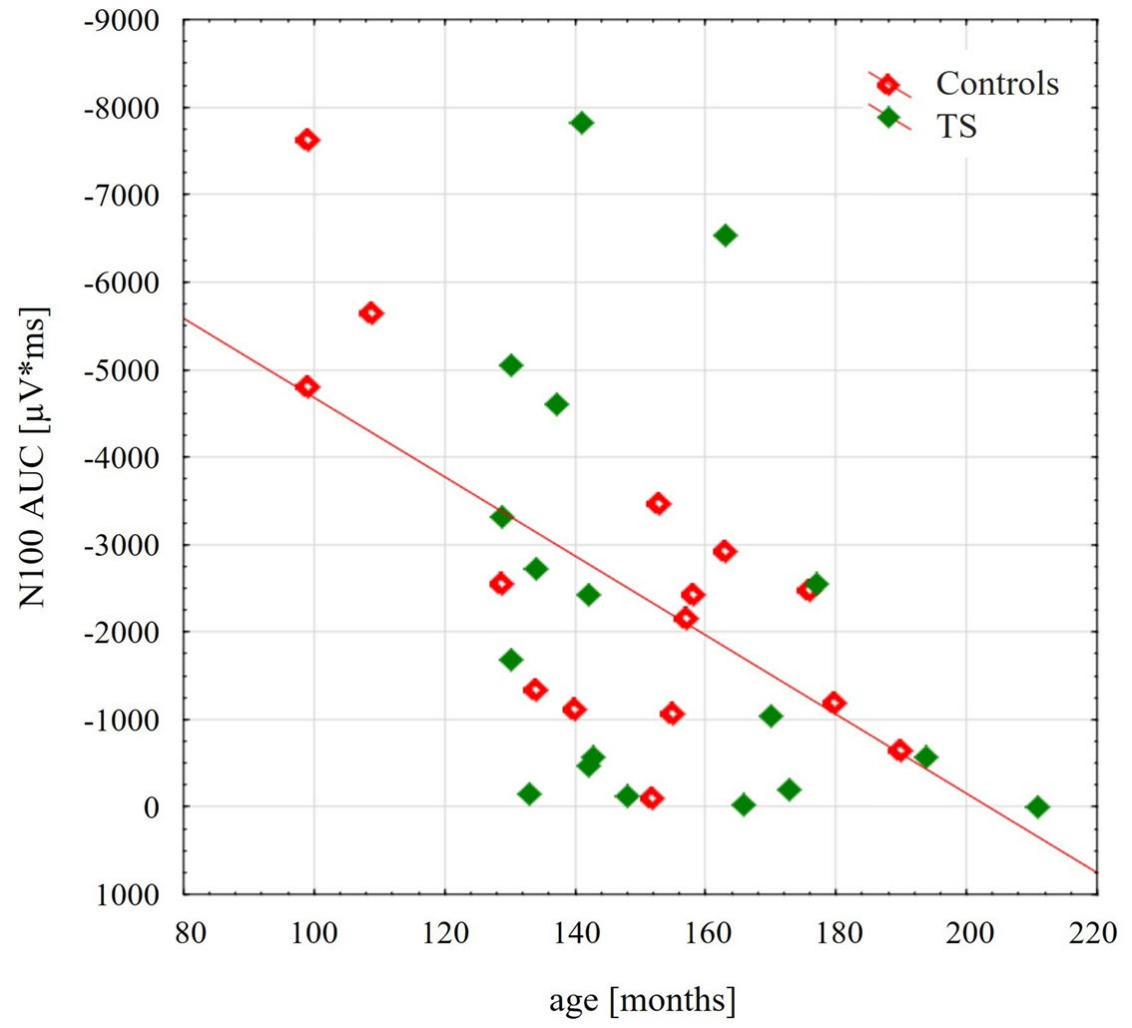
Scatterplot illustrating the age-related decrease of the TMS evoked N100 component, respectively, for control group (red) and for adolescents with tourette disorder (green). TEP-N100 amplitudes were recorded at CP6’ with a stimulation intensity of 110% resting motor threshold (RMT). Note that TEP-N100 component is represented as area under the curve (AUC) due to broad N100 potentials and high variability of N100 latency shown in children and adolescents.

TMS-evoked N100 amplitude at 110% RMT stimulation intensity during the resting condition did not differ between the groups [*F*(1;29) = 0.16; *p* = 0.69]. TMS-evoked N100 amplitudes showed an age-dependent maturational decrease with increasing age through late childhood and adolescence [*F*(1;29) = 13.4; *p* = 0.001], as shown in Figure 1. Although on a descriptive level, this decrease was more pronounced for control subjects, there were no significant differences between the two groups [interaction age × diagnosis *F*(1;29) = 0.45; *p* = 0.51].

**Figure 2:**
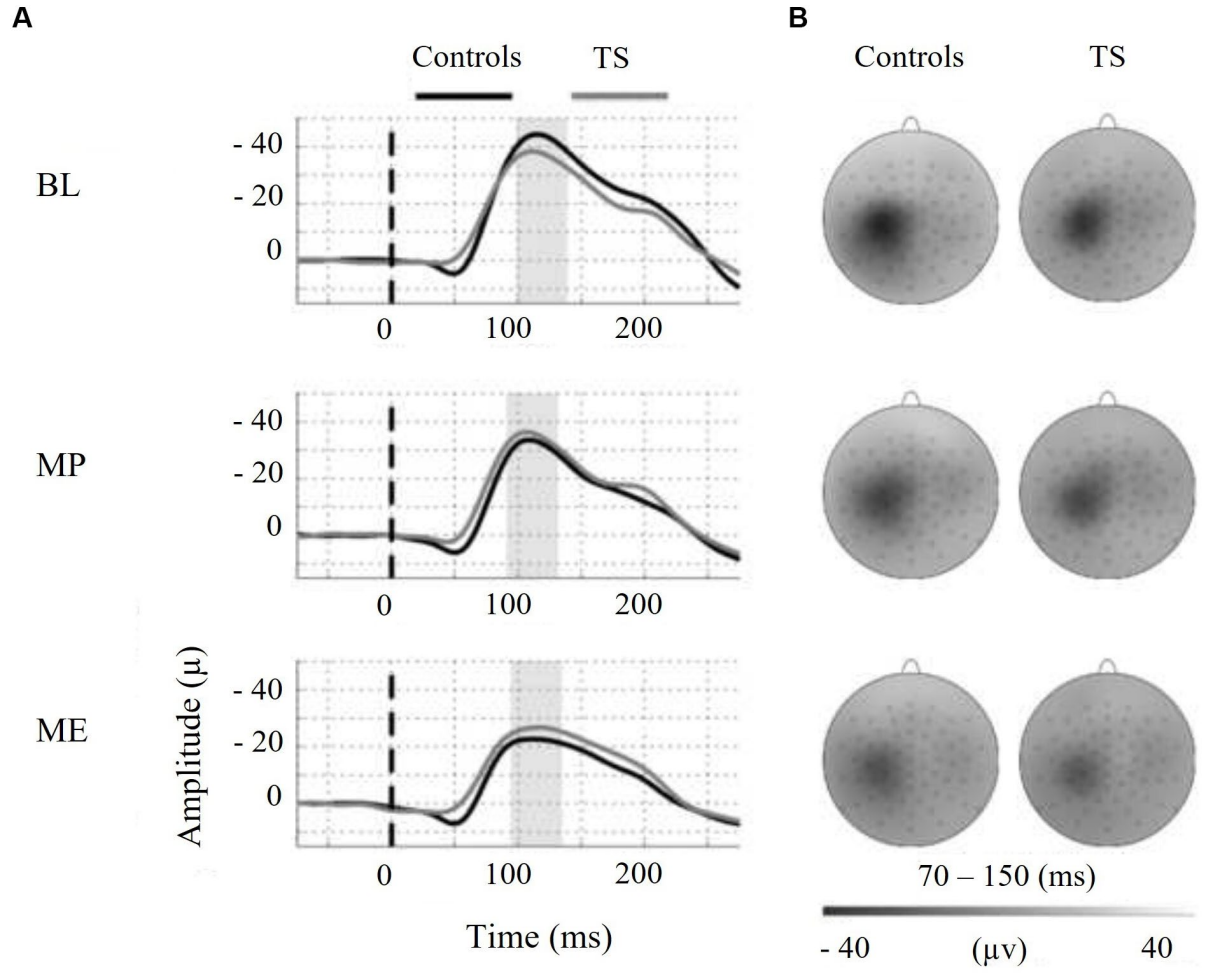
On the left-hand side, motor states are indicated as follows: BL = baseline, MP = motor preparation, ME = motor execution. **(A)** Shows the N100-TEP assessed at electrode C3. Note that voltage values at the y-axis are presented upside down. The vertical dashed line at time point zero indicates TMS, applied to the right motor cortex. The average N100-TEP amplitude latency by condition is highlighted in grey. **(B)** Shows voltage distribution around the TMS evoked N100 peak. The topographic time range selected represent the 95% confidence intervals of N100-TEP amplitudes across groups and conditions. Controls = typically developing subjects, TS = Tourette syndrome.

Moreover, we tested the internal modulation of N100 amplitude by movement preparation and movement execution (Table 2). Figure 2 shows the EEG response to TMS at electrode C3 averaged across control subjects and children with TS, respectively, for motor preparation as well as motor execution condition. Both conditions led to a significant reduction of TMS-evoked N100 amplitude compared to stimulation at rest [movement preparation *F*(1;29) = 6.9; *p* = 0.01; movement execution *F*(1;29) = 16.9; *p* = 0.0003], taking age and gender into consideration as covariates (main effects).

**Table 2:** Responsivity of inhibitory systems to different TMS intensities and internal modulation by movement state.

	40% MSO [μV*ms]	60% MSO [μV*ms]	80% MSO [μV*ms]
Controls	−84.0 ± 73.5	−307,9 ± 311.5	−2577.6 ± 2148.8
TS	−230.7 ± 320.1	−377,1 ± 520.8	−1908.3 ± 1454.3
	At rest (110% RMT) [μV*ms]	Motor preparation [μV*ms]	Movement execution [μV*ms]
Controls	−2632.7 ± 2046.5	−1921.0 ± 1810.9	−1158.1 ± 1380.3
TS	−2211.9 ± 2399.1	−1827.4 ± 1911.2	−1267.7 ± 1750.1

Please note that the mean of the individual differences between rest and motor states movement preparation and execution is NOT equal to the difference of the group means for these movement states, when comparing this table to Figure 2. TS = Tourette syndrome; MSO = maximum stimulator output.

**Figure 3:**
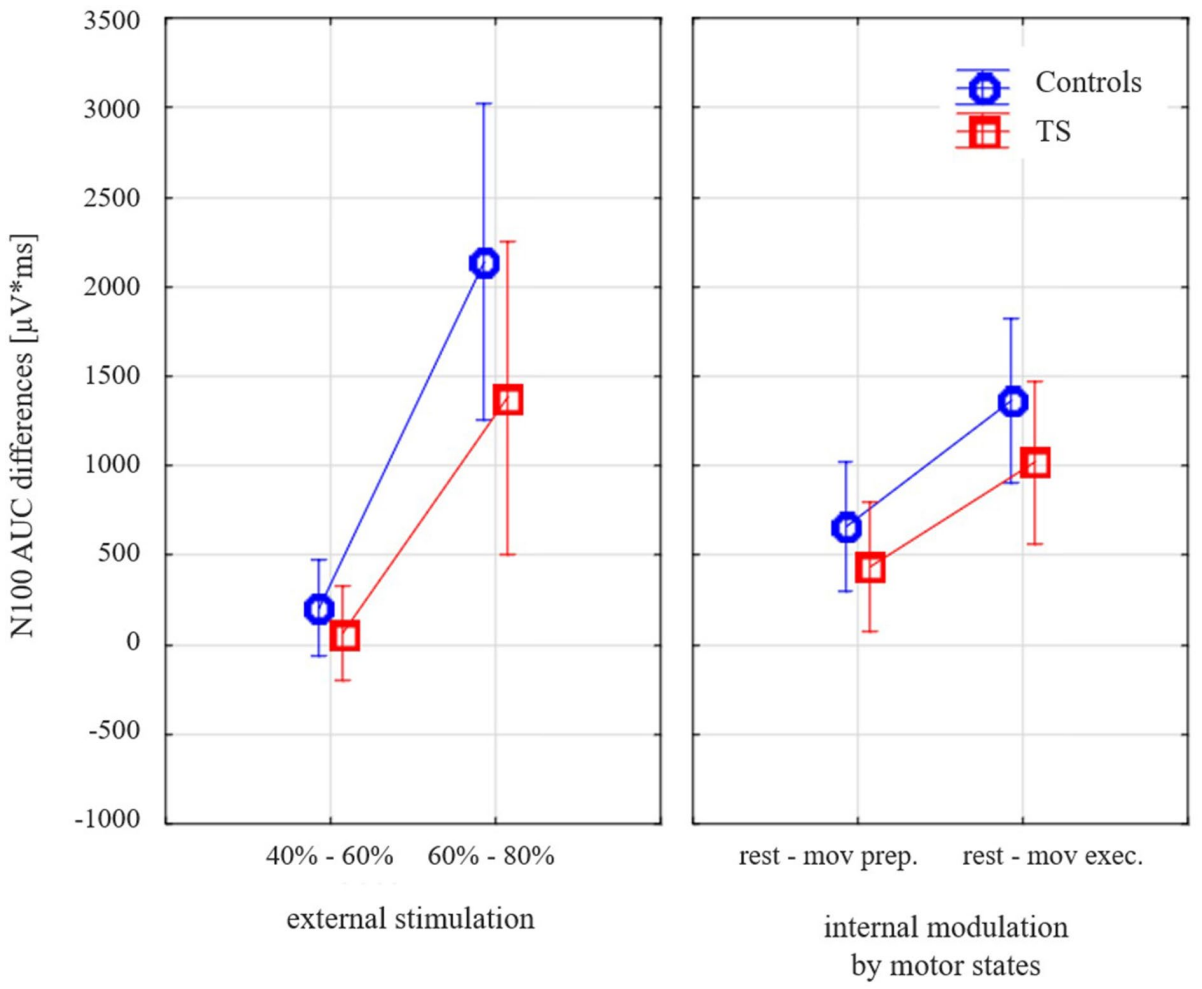
Effects of varying external stimulation intensity (increase from 40 to 60% maximum stimulator output, MSO, versus increase from 60 to 80% MSO) and internal modulation by motor state (difference resting state versus movement preparation and difference resting state versus movement execution) on the TMS-evoked N100 area under the curve (AUC) for subjects with Tourette syndrome and control subjects. Please note that all values illustrate adjusted means (after controlling for the effect of age and gender). Since the corrections for the covariates are taken into account, there are small deviations from the values in Table 2.

When the external and internal *modulation* of cortical inhibition (dependent variable TMS-evoked N100 amplitude *difference*) was examined (general linear model with the categorical predictors DIAGNOSIS (TS, CO), GENDER (female, male); the linear predictors AGE (months) and TMS-evoked N100 AREA UNDER THE CURVE (AUC) AT REST (110% RMT); the repeated measurement variables MODULATION TYPE (external stimulation versus internal modulation) and INTENSITY (small: stimulation difference 40 vs. 60% MSO, motor preparation, large: stimulation difference 60 vs. 80% MSO, motor execution)), subjects with TS showed less modulation than the control subjects [*F*(1;28) = 4.34; *p* = 0.047; Figure 3].

Controlling for age-dependent maturation (covariate age), this effect was similar for both types of modulation (different external stimulation intensities, internally prepared movement states) as there was no main effect of stimulation type [*F*(1;28) = 1.1; *p* = 0.30].

There was a strong effect of N100 amplitude at rest [*F*(1;28) = 15.4; *p* = 0.0005], which was stronger than simple age effects, justifying the inclusion of this covariate. There was no significant effect of comorbid ADHD [6/18 TS subjects; *F*(1;27) = 0.65; *p* = 0.43] or medication [3/18 subjects; *F*(1;27) = 0.12; *p* = 0.73], when included into the model, so these predictors did not enter the final model. In order to exclude artificial effects produced by covariates, we verified that there was still a strong trend towards the effect of diagnosis without suprathreshold TMS-evoked N100 amplitude at rest as a covariate [*F*(1;29) = 3.8; *p* = 0.06], with age now showing significant effects [*F*(1;29) = 7.4; *p* = 0.01].

### 3.3. Cortico-spinal-excitability

Resting motor thresholds did not differ between the two groups [*F*(1;29) = 0.49; *p* = 0.49], considering covariates age and gender. There was a trend towards a decrease of RMT with increasing age in both groups [*F*(1;29) = 2.9; *p* = 0.10].

When MEP amplitude at 110% was tested for group differences with the covariates age and gender, there was no significant difference between the groups [*F*(1;29) = 2.27; *p* = 0.14], despite descriptively lower amplitudes in children with TS (CO 389 ± 95 μV vs. TS 210 ± 55 μV).

## 4. Discussion

This study investigated potential differences in motor cortical inhibition (TMS-evoked N100 amplitude) in early adolescents with TS using combined TMS/EEG. We examined the modulation of inhibitory control in different activation states of the motor cortex. TMS/EEG studies of neurodevelopmental disorders are still rare. To our knowledge, this is the first study to report TMS evoked brain potentials (TMS-evoked N100, presumably related to GABA_B_-mediated cortical inhibition) in adolescent subjects with TS.

Our main findings are as follows: (1) TMS-evoked N100 amplitudes (cortical inhibition) at 110% RMT were comparable in early adolescent control subjects and subjects with TS; they showed no differences with respect to their cross-sectional maturational trajectory. (2) Compared to controls subjects, TS subjects showed reduced modulation of the GABA_B_-mediated TMS-evoked N100 when stimulated at varying fixed (non RMT-adjusted) intensities at rest and during top-down modulation by different motor states (movement preparation and movement execution). In sum, inhibitory systems in primary motor cortex were less responsive in TS. Reduced disinhibition of primary motor cortex from resting state to movement execution and reduced recruitment of GABA_B_-related inhibition in primary motor cortex to increasing intensities of external transcranial magnetic stimulation could point towards a reduced “inhibitory reserve” in the primary motor cortex (Heise et al., 2010). While the study of Heise et al. (2010) referred to a sample of 11 adult subjects with TS and short-interval intracortical inhibition (based on EMG-responses, GABA_A_-related), our study examined an early adolescent sample and used a cortical readout (TMS-evoked N100, GABA_B_-related). There was no main effect or interaction involving modulation TYPE. Thus, we obtained no hint in our data towards specific deficits in the two modulation TYPEs, however, we cannot exclude that such specific effect could be found in future studies in larger samples. Our conclusions refer to the capacity of modulation of motor cortex inhibition in general and not to either modulation type separately. Note that this concept of reduced modulation of inhibition in the motor cortex is different from higher cognitive control-related processes and inhibition of responses in a Go/NoGo task (such as reflected by a frontal N2 event-related potential component).

### 4.1. Motor cortical inhibition in subjects with TS

Impaired or altered inhibitory control has been proposed in many studies as a major cause of TS (Stern et al., 2008). However, there are also studies that did not find a deficient inhibitory performance (Ganos et al., 2014) or even an increased inhibitory control (Jackson et al., 2011).

Consistent with previous studies, we replicated the maturation related decline in GABA_B_-mediated TMS-evoked N100 amplitudes with increasing age (Bender et al., 2005; Bruckmann et al., 2012; D’Agati et al., 2014; Määttä et al., 2019). Moreover, our data showed that TMS-evoked N100 amplitudes decreases during motor preparation and execution, providing further evidence that TMS-evoked N100 represents motor cortical inhibitory processes. It has been shown that the TMS evoked N100 component as well as the TMS based long-interval intracortical inhibition (LICI) represent GABA_B_-receptor mediated neurotransmission (Farzan et al., 2013; Rogasch et al., 2013; Premoli et al., 2014). Therefore, the TMS-evoked N100 has been proposed to represent cortical mechanisms associated with GABA_B_-mediated motor cortical inhibition (Rogasch et al., 2013). Singer et al. (2001) showed that baclofen, a GABA_B_-agonist, did not lead to a reduction of tic symptoms in TS subjects. Our finding of normal TMS-evoked N100 amplitudes at 110% RMT could contribute to the notion that GABA_B_-mediated cortical inhibition in early adolescent TS might not be generally altered in TS motor cortex.

Compared to control subjects, TS subjects showed no significantly different resting motor thresholds as shown in other studies (Ziemann et al., 1997; Moll et al., 1999, 2001; Orth et al., 2005; Heise et al., 2010). However, when TMS was applied at progressive suprathreshold intensities, MEP recruitment curves were shallower in subjects with TS (Orth et al., 2008). The reported descriptively lower MEP amplitude at 110% would be in line with a shallower I/O curve. For most subjects, 40 and 60% MSO were subthreshold stimulation intensities, so no MEP changes corresponding to the TMS-evoked N100 could be obtained. Furthermore, motor cortex excitability was shown to be reduced in TS when examined at suprathreshold intensity during movement preparation (Heise et al., 2010; Draper et al., 2015) as well as movement execution (Jackson et al., 2013).

Concise, our data showed a reduced modulational effects of motor cortical inhibition in early adolescent subjects with TS for both motor states (movement execution more strongly than movement preparation) and increasing external stimulation, leading to a shallower stimulus-intensity slope of the TMS-evoked N100 component. These findings corroborate to the notion that the responsivity and recruitment of synaptic inhibition is deficient to both top-down modulation and external stimulation in early adolescent TS whereas axonal excitability is normal (Orth et al., 2005). Differences of control subjects and TS subjects may arise due to differences in balancing between motor cortical excitatory and inhibitory processes. Orth et al. (2005) found a reduced inhibitory interaction between sensory input and motor output in TS. They assumed that sensory input could lead to a reduction of motor cortical output in order to prevent involuntary movements. Therefore, a reduced disinhibition during distinct motor states and in response to external stimulation could represent a mechanism to reduce the triggering of tic movements. Differences of cortical inhibition might arise due to a divergent input from multiple sites within the cortico-basal ganglia-thalamocortical circuit to the primary motor cortex. A recent hypothesis classifies tics rather as a surplus of action due to an abnormally strong perception-action binding (Beste and Münchau, 2018). Our finding that modulation of motor cortex inhibition by top-down control and by external stimulation was reduced in TS, would be well compatible with increased perception-action binding, i.e., a control of motor cortex excitability by other sources than within the motor system. A reduced top-down modulation of motor cortex excitability could be seen as contributing to this strong perception-action binding due to relatively stronger bottom-up than top-down control. However, the similar effect of varying movement related brain states and stimulation intensity rather points towards a reduced motor inhibitory reserve to any kind of modulation within the motor system. In any case, our findings are well compatible with increased perception-action binding and point towards a specific contribution of the developing motor system.

So far, mechanisms underlying altered top-down modulation of motor cortical inhibition in TS have not been well understood, likely because both, short- and long-range cortical patterns of cortical connectivity may be involved. Various motor cortical excitability measures in TS lead to the assumption that all motor cortical circuits may show a reduced gain (for reviews, see Orth, 2009; Orth and Münchau, 2013). Many recent studies have focused specifically on the role of the supplementary motor area (SMA) with regard to TS pathophysiology and showed the following: First, the SMA shows increased activity immediately before the onset of a tic (Bohlhalter et al., 2006). Second, functional connectivity between SMA and motor cortex is increased in TS subjects compared to control subjects (Franzkowiak et al., 2012). Third, inhibitory repetitive TMS applied to the SMA caused a decrease in tic frequency (Mantovani et al., 2006; Kwon et al., 2011; Le et al., 2013). Fourth, using magnetic resonance spectroscopy, Draper et al. (2014) recently showed that GABA concentration related to SMA was increased in TS subjects compared to controls. Moreover, GABA concentration within the SMA was inversely associated with motor cortex excitability. The altered modulation of the TMS evoked N100 component during internal modulation by movement states could be caused by “upstream” modifications in the SMA. However, we also observed an altered modulation of the TMS-evoked N100 when the primary motor cortex was stimulated at rest with different intensities. This suggests either a deficit directly within the primary motor cortex or a tonic effect of SMA inputs or other circuits (e.g., including the basal ganglia) on the primary motor cortex at rest. From our data, we cannot infer which other cortical or subcortical areas may be involved in reduced efficiency of motor cortical inhibition in TS or which subcortical or cortical areas may act as “tic generators,” creating unintended motor system excitation and triggering of tic movements.

Even though our TS sample did not exhibit a uniform operationalization of motor states regarding movement preparation and initiation in comparison to previous studies, the most striking distinction appears to be the lower age range of the investigated TS sample. Many TS subjects gain control over their tics during adolescence and experience symptom relief reaching adulthood. It is presumed, that compensatory changes in brain structure and function of adolescent TS subjects lead to an elevated tonic inhibition which in turn improve the control over motor output (Plessen et al., 2004; Serrien et al., 2005; Mueller et al., 2006; Jackson et al., 2011; Jung et al., 2013). However, in our study, we investigated a reduced responsiveness of the motor cortex rather than a generally increased cortical inhibition. The age of the investigated TS sample could play a major role regarding reported differences in TS related mechanisms. Since TS shows an age-related development reaching the maximum severity of symptoms in early adolescence (Bloch and Leckman, 2009), compensatory mechanisms may adapt to developmental changes of underlying deficits. It is conceivable that a reduced motor cortical output as a response to external input is no longer sufficient to control involuntary movements effectively and may need to be replaced or extended by an overall elevated tonic inhibition.

### 4.2. Limitations

It should be noted that some confounding influences may not be completely ruled out regarding the TMS-evoked N100 component, such as the sound produced upon TMS pulse emission. Although all participants wore earplugs to minimize sensory confounds, it has been shown that auditory evoked potentials (AEP) are nonetheless at least partially superimposed upon the TMS-evoked N100 amplitude (Ter Braack et al., 2015). Besides AEPs, also SSEPs can have an influence on TEPs. However, previous studies investigated that early SSEP components occur with a latency of around 20 ms following the pulse (Verroust et al., 1990). Pokorny et al. (2022) showed, that late SSEP components are most prominent over contralateral somatosensory areas, in contrast to the analyzed N100 potential (ipsilaterally to the stimulation). In this context, it has to be considered that most patients with TS experience sensory hypersensitivity to internal and external stimuli, due to altered central processing of perceptual information, including auditory stimuli (Kleimaker et al., 2020). Therefore, it cannot be ruled out, that AEPs and SSEPs had a divergent influence on the TMS evoked N100 component of the analyzed groups. However, the analysis of broad TMS-evoked N100 areas under the curve should have minimized possible AEP and SSEP influences. Moreover, children show a reduced auditory N100 component projecting from the auditory cortex to central areas (Bender et al., 2005). No ICA components with characteristic AEP topography could be detected, confounding our data. AEPs show a lower amplitude, less duration and less lateralized potentials than the TMS-evoked N100 component reported here.

## 5. Conclusion

Single-pulse TMS was used to assess alterations in motor cortical excitability during resting condition, movement preparation and movement execution in young adolescents with TS. Our data showed a reduced cortical responsiveness of TS subjects to external stimulation by TMS and a reduced modulational effects of movement related brain states on motor cortical inhibition, compared to control subjects. These results provide preliminary evidence of altered modulation of motor cortical inhibition related to GABA_B_-mediated inhibitory processes and show evidence for a reduced efficiency of the primary motor cortical inhibition (reduced “inhibitory reserve”) in TS.

## Data availability statement

The datasets presented in this article are not readily available because participants have not consented to the transfer of data. Requests to access the datasets should be directed to julia.schmidgen@uk-koeln.de.

## Ethics statement

The studies involving humans were approved by Ethics Committee at the TU Dresden. The studies were conducted in accordance with the local legislation and institutional requirements. Written informed consent for participation in this study was provided by the participants’ legal guardians/next of kin.

## Author contributions

JS contributed to the main data analysis, to interpretation of the results and to manuscript writing. SB contributed to both the conception and design of the study, provided supervision for data analysis, and edited the manuscript. KK and VR contributed to manuscript revision. All authors contributed to the article and approved the submitted version.

## Abbreviations

ADHD: Attention deficit hyperactivity disorder
AEP: Auditory evoked potential
CNV: Contingent negative variation
CSTC: Cortico-striato-thalamocortical
EEG: Electroencephalogram
EHI: Edinburgh Handedness Inventory
GABA: γ-aminobutyric acid
LICI: Long-interval intracortical inhibition
MEP: Motor evoked potential
MSO: Maximum stimulator output
RMT: Resting motor threshold
SMA: Supplementary motor area
TS: Tourette syndrome
TEP: TMS evoked potential
TMS: Transcranial magnetic stimulation
YGTSS: Yale Global Tic Severity Scale
